# Socio-Gerontechnology – ein Forschungsprogramm zu Technik und Alter(n) an der Schnittstelle von Gerontologie und Science-and-Technology Studies

**DOI:** 10.1007/s00391-021-01862-2

**Published:** 2021-03-03

**Authors:** Anna Wanka, Vera Gallistl

**Affiliations:** 1 Goethe Universität Frankfurt, Frankfurt am Main, Deutschland; 2 Institut für Soziologie, Universität Wien, Wien, Österreich; 3 Karl-Landsteiner Universität für Gesundheitswissenschaften, Krems, Österreich

**Keywords:** Techniknutzung, Digitalisierung, Partizipatives Design, Ko-Konstitution, Kulturgerontologie, Technology use, Digitalization, Participatory design, Co-constitution, Cultural gerontology

## Abstract

**Hintergrund:**

In der Gerontologie beschäftigt sich Forschung intensiv mit der Frage, wie und wieso ältere Menschen neue Technologien nutzen oder nicht. Diese Forschung basiert mehrheitlich auf einem differenzierten Alter(n)sverständnis, verwendet jedoch einen verengten Technikbegriff, der Technologien auf manifeste Artefakte reduziert. Zur Erweiterung ihres Technikbegriffs kann die Gerontologie von den Science-and-Technology Studies (STS) lernen. Deren Ansätze und Perspektiven auf Technik und Alter(n) werden im Beitrag diskutiert, und es wird der Frage nachgegangen, wie anschlussfähig solche an aktuelle gerontologische Debatten und Befunde sind.

**Material und Methode:**

Auf Basis aktueller Literatur wird in diesem Beitrag 2 Fragen nachgegangen: Welche Aspekte werden aus einer STS-Perspektive im Themenbereich Alter(n) und Technik thematisiert? Welche konzeptionellen Positionen zeichnen eine Forschungsperspektive der STS auf Technik und Alter(n) aus?

**Ergebnisse:**

Im Themenbereich Alter(n) und Technik beschäftigen sich STS-Studien einerseits mit Entwicklungs- und Designprozessen neuer Technologien für ältere Menschen und andererseits mit den alltagsweltlichen Interaktionen zwischen Technologien und älteren Menschen.

**Schlussfolgerungen:**

Auf Basis der Ergebnisse skizziert der Beitrag das Feld der Socio-Gerontechnology als Forschungsprogramm an der Schnittstelle zwischen STS und Gerontologie anhand von 3 Themen: (1) Materialitäten des Alter(n)s, die über innovative Technologien hinausgehen, (2) das Verhältnis von Alter(n)s- und technologischen Innovationsdiskursen und (3) die technologische Handlungsmächtigkeit von älteren Menschen.

## Abstract (en)

**Background:**

A vast body of gerontological research addresses the questions how and why older adults use new technologies or not. While the majority of them advocate a differentiated understanding of age(ing), most are based on a narrow understanding of technology, reducing it to manifest artefacts. To broaden such an understanding, gerontology can learn from science and technology studies (STS). This article discusses STS approaches and perspectives on age(ing) and technologies as well as their compatibility with gerontological research.

**Material and methods:**

Based on state of the art literature in STS, this research overview addresses two questions: which aspects does an STS perspective on age(ing) and technologies emphasize? Which concepts are being used in STS to research age(ing) and technologies?

**Results:**

Respective research analyzes firstly, how (negative) images of ageing are inscribed into technologies throughout the design and development process and secondly, the creative everyday interactions between older adults and technologies. In doing so, images of ageing, technological artefacts, developers and older (non)users are equally conceptualized as agential.

**Conclusion:**

The paper outlines a socio-gerontechnological research agenda by outlining three thematic areas: (1) materialities of ageing that exceed innovative technology, (2) the relations between images of ageing and technological innovation discourses, and (3) the technological agency of older adults.

Neue Technologien werden zunehmend speziell für ältere Menschen entwickelt. Trotz der Beständigkeit, mit der Fördergeber*innen die Entwicklung und Implementierung von Alterstechnologien (etwa durch das europäische Ambient/Active Assisted Living Joint Programme, AAL-JP) fördern, haben sich diese bislang nicht auf dem Markt durchgesetzt [11]. Das liegt u. a. daran, dass Forschung und Entwicklung die Einbettung von Technologien in den Alltag älterer Menschen zu wenig berücksichtigen und Altersbilder nur unzureichend reflektiert werden [18].

## Gerontologische Forschung zu Technik und Alter(n)

Digitale Technologien^1^ sind zu Beginn des 21. Jh. untrennbarer Bestandteil der Lebenswelten älterer Menschen. Die technische Entwicklung und der demografische Wandel stehen zunehmend in einem Wechselverhältnis: Der demografische Wandel wird – vor dem Hintergrund der Entwicklung assistiver Technologien – zunehmend durch (digitale) Technologien gestaltet; gleichzeitig findet der technische Wandel vor dem Hintergrund sich wandelnder Altersstrukturen westlicher Gesellschaften statt. So zeigt sich etwa im jährlichen Digitalindex der Initiative D21, dass 2018 mehr als drei Viertel der 60- bis 69-Jährigen und knapp die Hälfte der 70+-Jährigen in Deutschland das Internet nutzten und viele (55 resp. 24 %) dies auch mobil, etwa über das Smartphone oder Tablet, tun [5]. Aufgrund der zunehmenden Relevanz dieser beiden Entwicklungen entstand an der Schnittstelle von gerontologischen und technikwissenschaftlichen Fragestellungen in den letzten Jahren das lebhafte und vielfältige Forschungsfeld der Gerontechnologie, das sich durch Interdisziplinarität und eine Orientierung an angewandter Forschung auszeichnet [18].

In der Gerontologie hat die technische Entwicklung in den letzten Jahren 3 Diskussionsstränge und Forschung angestoßen [32]: erstens Forschung darüber, wie Technologien besser zu einem selbstbestimmten Altern beitragen können, im Feld der (funktionalistischen) Gerontechnologie. Hier fokussieren Studien beispielsweise auf Modelle und Interventionen im Bereich der Technikakzeptanz [21]. Zweitens findet sich in der Gerontologie Forschung zu Risiken sozialer Exklusion durch unzureichende Nutzungsmöglichkeiten neuer Technologien für ältere Menschen im Feld der kritischen Sozialgerontologie. Hier fokussieren Studien beispielsweise auf Möglichkeiten zur Unterstützung digitaler Teilhabe oder den Aufbau digitaler Kompetenzen [6], und drittens findet sich Forschung zu Risiken negativer Stereotypisierung und Standardisierung des Alter(n)s durch die Entwicklung altersspezifischer Technologien im Feld der kritischen Kulturgerontologie, die sich beispielsweise damit beschäftigt, wie neue Technologien das Alter(n)serleben beeinflussen [20]. Die gerontologische Forschung hat dadurch einen reichen und vielfältigen Wissensbestand über Fragen von Techniknutzung im Alter generiert.

Allerdings zeigen sich auch blinde Flecken gerontologischer Forschung zu Technik und Alter(n): So teilt die Gerontologie zwar großteils ein differenziertes Verständnis vom Alter(n), jedoch ein weniger differenziertes Verständnis von Technik [30]. Die Erweiterung eines solchen gerontologischen, „engen“ Technikbegriffs ist ein Anliegen von Forschung zu Technik und Alter(n) außerhalb der Gerontologie, v. a. in den Science-and-Technology Studies (STS) geworden.

## Perspektiven der STS auf Technik und Alter(n)

Ähnlich wie die Gerontologie sind die STS ein interdisziplinäres und vielfältiges Forschungsfeld, das sich mit Wissenschaft und Technik aus einer sozialwissenschaftlichen Perspektive beschäftigt [8] und das Technik und Gesellschaft nicht als zwei getrennte Bereiche, sondern als relational und kokonstitutiert versteht [15].

In Bezug auf das Alter(n) sprechen Joyce und Mamo [16] hier, aufbauend auf dem STS-Konzept des „Cyborgs“ als Mischwesen menschlicher und nichtmenschlicher Elemente [13], von einem „ergrauenden Cyborg“. Dieser Begriff beschreibt, dass Technologien in digitalisierten Gesellschaften nicht als bloße Instrumente von älteren Menschen genutzt, akzeptiert oder abgelehnt werden, sondern dass sie im Zusammenspiel mit menschlichen Körpern das Alltags- und Alterserleben signifikant mitbestimmen. Sie bedingen somit, wie alt wir uns subjektiv fühlen und als wie alt wir sozial adressiert werden – etwa, wenn die Kompetenzen fehlen, ein neues technisches Gerät zu nutzen oder wenn eine assistive Technologie, z. B. ein Hörgerät, sichtbar zur Alterskategorisierung beiträgt. STS, die sich mit dem Alter(n) beschäftigt, untersucht also, wie Technologien die Bedeutungen und Erfahrungen des Alter(n)s mitbeeinflussen und hervorbringen, und welche Rolle ältere Menschen in diesem Prozess spielen (z. B. [27]).

Wie anschlussfähig sind Forschungsarbeiten der STS an gerontologische Debatten? Anhand von aktueller Forschungsliteratur aus der STS geht der Artikel dieser sowie zweier spezifizierender Fragen nach:

1. Welche Aspekte werden aus einer STS-Perspektive im Themenbereich Alter(n) und Technik thematisiert?
2. Welche konzeptionellen Positionen zeichnen eine Forschungsperspektive der STS auf Technik und Alter(n) aus?

Die im Folgenden dargestellten Forschungsarbeiten wurden ausgewählt, weil sie die zugrunde liegenden Prämissen der STS zu Themenbereichen des Alter(n)s besonders gut verdeutlichen. Um die theoretische Tiefe dieser Arbeiten darstellen zu können, wurde bewusst auf eine systematische Literaturübersicht verzichtet.

### Welche Aspekte werden aus einer STS-Perspektive im Themenbereich Alter(n) und Technik thematisiert?

In der von Konzepten der STS inspirierten Forschung zu Alter(n) und Technik findet sich eine thematische Zweiteilung [28]: Erstens wird auf den Technikentwicklungsprozess fokussiert und danach gefragt, wie Altersbilder und -diskurse, etwa jene des „aktiven Alterns“ oder des „ageing in place“, in Gerontechnologien eingeschrieben werden (z. B. [7, 24]). Eine zentrale Debatte stellt hier die kritische Betrachtung von Nutzer*inneneinbindung in der Technikentwicklung dar: In einem Literaturreview zu diesem Thema stellten etwa Fischer et al. [10] fest, dass es kaum Evidenz dafür gibt, dass eine solche die spätere Technikakzeptanz bei älteren Menschen erhöht. Dies liege, so die Autor*innen, teilweise an den marktwirtschaftlich getriebenen Motivationen für die Einbindung älterer Menschen, den Machtungleichheiten zwischen Entwickler*innen und älteren Menschen, den Verzerrungen bei der Stichprobengenerierung, den negativen Altersbildern, die sich darin manifestieren, und der fehlenden Offenheit dieser Prozesse.

Ein Beispiel hierfür stellt die Pflegerobotik dar, die trotz intensiver Förder- und Entwicklungsbemühungen nicht über den Projektstatus hinauskommt [17]. Den Grund dafür verorten Forscher*innen der STS in den dahinterliegenden Bedingungen partizipativer Technikentwicklung, die sich nicht aus Idealen guter Pflege, sondern aus der Verschaltung von politischen, ökonomischen und technologischen Innovationsidealen entwickelt haben. Obwohl Technikentwicklungsprogramme der Pflegerobotik explizit Vorgaben zur nutzer*innenzentrierten Gestaltung enthalten, wird die Entwicklung meist von technischen Möglichkeiten begrenzt, während Fragen rund um ein lebenswertes Alter(n) und guter Pflege in den Hintergrund treten [3].

Als zweites Forschungsthema widmet sich STS-Forschung zu Alter(n) und Technik den alltäglichen Nutzungspraktiken von Technologien durch ältere Menschen. So untersuchen etwa Fernandez-Ardevol et al. [9] Unterschiede und Gemeinsamkeiten in Smartphone-Nutzungspraktiken bei älteren Menschen in Spanien, den Niederlanden, Schweden und Kanada. Eine zentrale Rolle wird dabei der Handlungsmacht sowohl aufseiten der Technik als auch aufseiten der älteren Nutzenden zugeschrieben: *Older individuals, rather than being passive users of (digital) technology, play an active role by domesticating reconfiguring, modifying or rejecting it in their everyday life *[9, S. 49]. Die kreative, von Designer*innen nichtintendierte, Nutzung von Technologien spielt innerhalb dieses Forschungsstrangs eine besondere Rolle. Berschold et al. [2] stellen etwa fest, dass Gerontechnologien häufig an den Bedürfnissen und Lebenswelten älterer Menschen vorbeigehen und es dadurch notwendig machen, dass ältere Menschen diese *anders *– nämlich an ihren eigenen Alltagspraktiken und Präferenzen ausgerichtet – nutzen. Diesen Prozess beschreiben sie als „innosumption“ [28], wodurch sie ältere Menschen nicht nur als Nutzende, sondern auch als Designer*innen von Technologien verstehen. Technologien werden aus einer solchen Perspektive durch die alltägliche Nutzung von älteren Menschen erst zu einem spezifischen Instrument gemacht, d. h. durch Nutzungspraxis, die Kombination mit anderen (technischen) Geräten und Arrangements (weiter-)entwickelt. Dadurch werden auch nichtintendierte Nutzungspraktiken älterer Menschen als aktiv und kreativ, also schöpferisch, anstatt potenziell inkompetent und fragil, gerahmt [12].

### Welche Positionen zeichnen eine STS-Perspektive auf Technik und Alter(n) aus?

Forschung an der Schnittstelle von STS und Gerontologie positioniert sich meist vor dem Hintergrund einer geteilten Gesellschaftsdiagnose: Der demografische Wandel wird in westlichen Gesellschaften als Problem verstanden, für das technologische Innovation eine mögliche Lösung darstellt [22, 27, 28], und Forschungsarbeiten in der STS versuchen, diese Prämissen kritisch zu hinterfragen. Als Basis für solche Prämissen werden einerseits ein breiterer Biomedikalisierungsdiskurs um den alternden Körper, in dem Alter(n) an sich als Krankheit verstanden wird, und andererseits die Entstehung einer Gerontechnologie-Industrie, die sich jedoch nicht in den Lebenswelten älterer Menschen durchsetzt [16, 25, 26], genannt. Als ein Grund dafür wird eine interventionistische und paternalistische Ausrichtung der derzeitigen Technikforschung und -entwicklung ausgemacht [25, 27]. Peine argumentiert dabei:

*it may be necessary to abandon the widespread interventionist vocabulary that haunts current debates around ageing, health and technology, and that is expressed in terms like „impact,“ „solution,“ or „acceptance.“ For sure, these terms have their merits […] but they also assume, in one way or another, that there are stable and measurable effects of technologies on the lives of people and patients. *(Peine [25, S. 666])

Trotz der zunehmenden Digitalisierung wird der derzeitigen Technikgerontologie zugeschrieben, *both under-theorized and over-instrumentalized *[25] zu sein. Dabei wird aus einer STS-Perspektive ein überwiegend instrumentelles Technikverständnis – Technik als Instrument zur Lösung von Problemen – ebenso kritisiert wie ein überwiegend passives, defizitorientiertes Altersbild, das ältere Menschen auf inkompetente, häufig technikaversive „user“ reduziert [24]. Stattdessen schlägt eine STS-Perspektive vor, Alter(n) und Technik als relationale Konzepte zu verstehen, die sich gegenseitig konstituieren.

*With the notion of co-constitution … we suggest an approach that is attentive to the many ways in which the experience of ageing itself is constituted together with the increasing diffusion of technological innovations. … studies in sociogerontechnology have shown how technological innovation creates ageing and older people as much as it targets them.* (Peine et al. [28])

Eine solche Perspektive geht also im Kern davon aus, dass Alter(n), Technologien und die sozialen Kontexte, in die diese eingebettet sind, untrennbar miteinander verbunden sind [7, 9, 14, 19]. So zeigen empirische Forschungen aus den STS, wie Altersdiskurse im Prozess der Technikentwicklung in neue Technologien eingeschrieben werden („age scripting“) und die Interaktion mit diesen Technologien das eigene Alterserleben verändert [5]: Wird etwa bei der Entwicklung von Sensoren nicht mitbedacht, dass ältere Menschen sexuell aktiv sind und sexuelle Praktiken dadurch evtl. einen Alarm auslösen, kann das die Vermeidung solcher Praktiken und ein verändertes Alterserleben zur Folge haben [31]. In einem Prozess des „Entskriptens“ („descripting“) werden diese Vorstellungen von älteren (Nicht‑)Nutzer*innen in Forschung, Entwicklung und im Alltag reflexiv gemacht, verhandelt und umgedeutet.

Von einer derartigen Kokonstitutionsprämisse auszugehen, setzt voraus, sowohl Technologien als auch älteren Menschen relevante Handlungsmächtigkeit („agency“) zuzuschreiben. Daraus ergibt sich für die empirische Forschung, der Fragestellung nachzugehen, *wie* – d. h. durch welche soziomateriellen Praktiken – sich Alter(n) und Technik kokonstituieren, anstatt einen vermeintlichen Einfluss von Techniknutzung auf messbare Parameter des Alter(n)s (z. B. Gesundheit, soziale Kontakte) zu untersuchen [23].

## Diskussion

Auf Basis der Literatur wurden Unterschiede zwischen Forschungsarbeiten in der Gerontologie [27] und Perspektiven der STS auf Technik und Altern deutlich: Während die Gerontologie ältere Menschen in den Fokus ihrer Betrachtungen stellt, geht es der STS primär um Technologien. Beide Felder teilen aber auch Erkenntnisinteressen, etwa, wie sich neue Technologien in den Alltag älterer Menschen integrieren lassen, und wieso bestimmte Technologien von älteren Menschen weniger genutzt werden.

Einen entscheidenden Schritt zur Weiterentwicklung gerontologischer Forschung geht die STS in ihrem Technikverständnis, das sich deutlich von instrumentellen Technikbegriffen, die Forschung in der Gerontologie häufig zugrunde liegen [18], unterscheidet. Technologien und Altern, so die Perspektive der Kokonstitution innerhalb der STS [9, 25], sind untrennbar miteinander verbunden. Technologien sind in einer digitalisierten Welt nicht nur Instrumente, sondern aktive und gestaltende Akteur*innen im Alltagsleben aller älterer Menschen – und nicht nur jener älterer Menschen, die sie auch nutzen.

Wie anschlussfähig sind STS-Debatten an die einleitend skizzierten Diskussionen in der gerontologischen Forschung [18]? Erstens zeigt sich, dass Konzepte der STS die Forschung zur Einbettung von Technologien in den Alltag älterer Menschen, und damit auch ihrer Nutzung und Wirkung, erweitern können [9, 16]. Forschung mit einem STS-Vokabular und -Instrumentarium geht alltagsnah an die Lebenswelten älterer Menschen heran und fragt danach, welche Prozesse eine Einbettung von Technologien in den Alltag älterer Menschen bedingen. Beispiele eines solchen Vokabulars sind der Begriff der Soziomaterialität des Alter(n)s [16], der darauf hinweist, dass Lebenswelten älterer Menschen immer von sozialen und materiellen Aspekten gekennzeichnet sind; Konzepte des Age script oder des Descripting [7], mit denen beschrieben werden kann, wie sich Vorstellungen über ältere Menschen in Technologien einschreiben und umgedeutet werden können; oder der Begriff der Innosumption [28] der ältere Menschen als Nutzende und als Designer*innen von Technologien versteht.

So eine Perspektive ermöglicht es nicht (nur) zu fragen, ob und inwiefern Technologien und Techniknutzung positiven oder negativen Einfluss auf ältere Menschen nehmen, sondern auch, wie ein solcher Einfluss zustande kommt [26]: Welche Diskurse und Problemstellungen werden durch neue Technologien transportiert, und wie hängen diese mit ihren (Nicht‑)Nutzungsweisen zusammen? Welche Probleme sollen Technologien lösen, und sind diese aus Perspektive älterer Menschen Probleme, die durch Technik zu lösen sind? Welche nichtintendierten Nutzungspraktiken und Formen der Nichtnutzung finden sich, und wie hängen diese mit der Lebensqualität älterer Menschen zusammen? Die STS trägt damit zu einer konzeptionellen und methodischen Weiterentwicklung einer funktionalistischen gerontologischen Forschung zu Alter(n) und Technologien bei, die sich mit dem Effekt von Techniknutzung im Alter beschäftigt.

Zweitens zeigt sich, dass Konzepte der STS die Diskussion rund um digitale Exklusion älterer Menschen erweitern, weil sie durch ihren Fokus auf Rahmenbedingungen der Technikentwicklung darauf hinweist, wie diese Exklusion zustande kommt. Technik und Alter(n), so zeigt Forschung in der STS auf, findet in einem Feld statt, in dem Fördergeber*innen, Technikentwickler*innen und ältere Menschen in bestimmten Strukturen verbunden sind und ältere Menschen meist eine marginalisierte Position innehaben [32]. Forschungsarbeiten innerhalb der STS erweitern Forschungsarbeiten der kritischen Sozialgerontologie zu Technik und Alter(n), da sie den Fokus nicht nur auf den exkludierenden Charakter von Technologien, sondern auch auf den exkludierenden Charakter von Technikforschung und -entwicklung, sowie den systemischen und strukturelle Mechanismen im Feld der Technikentwicklung, legt.

Drittens zeigt sich, dass eine STS-Perspektive kulturgerontologische Forschung befruchten kann. Autor*innen der STS (z. B. [14, 28]) schlagen Brücken zur Kulturgerontologie und zur qualitativen Gerontologie, indem sie die soziale Konstruktion des Alterns [1, 4] mit einer materialistischen STS-Perspektive ergänzen: Alter(n) wird dabei nicht nur als soziale und kulturelle, sondern soziomaterielle Konstruktion verstanden [14]. In digitalisierten Umwelten und Kulturen, so die Argumentation, finden Konstruktionen des Alter(n)s immer materiell eingebettet, d. h. durch Bezugnahme auf bestimmte Technologien, statt. Hier sind Konzepte aus der STS v. a. dort anschlussfähig, wo in der Gerontologie mit qualitativer, alltagsnaher Methodologie an den Forschungsgegenstand Alter(n) und Technik herangegangen wird [29].

Wie könnte eine Forschungsagenda in der Schnittmenge zwischen STS und Gerontologie aussehen? Die Literatur skizziert 3 Bereiche eines solchen Forschungsprogramms der „Socio-Gerontechnology“: erstens Forschung zu den Materialitäten des Alter(n)s, die sich mit der soziomateriellen Konstitution des Alter(n)s beschäftigt und die Rolle technischer Materialitäten – vom Fitnessarmband zum Sensorboden, zur Waschmaschine – im Alltagsleben älterer Menschen in den Blick nimmt. Dabei sollten der Blick auf innovative Technologien erweitert werden und auch jene Technik berücksichtigt werden, die bereits im Alltagsleben älterer Menschen verankert ist. Zweitens skizziert das Forschungsprogramm der Socio-Gerontechnology das Forschungsthema der Technik- und Altersdiskurse. Anknüpfend an die bestehende gerontologische Forschung zu Altersbildern könnten Innovationsdiskurse in den Fokus der Gerontologie rücken und auf „ageism“ geprüft werden. Drittens und letztens hebt ein Forschungsprogramm der Socio-Gerontechnology das Forschungsthema der Handlungsmächtigkeit durch und mit digitalen Technologien hervor. Noch stärker als bisher sollte Forschung zu Alter und Technik die Handlungsmächtigkeit älterer Menschen fokussieren und so unerwartete, nichtintendierte Nutzungsweisen nicht als Fehlnutzung, sondern als „agency of older people in negotiating a meaningful space for technology in their lives“ [27] verstehen [2]. Derartige Befunde können als Stimulus für Designprozesse fungieren, in denen insbesondere die Nutzer*inneneinbindung ein bedeutsames zukünftiges Forschungsfeld der Socio-Gerontechnology darstellt.

## Praxisimplikationen

- Für Technikentwickler*innen zeigt die STS auf, worauf bei der partizipativen Entwicklung von Alter(n)stechnologien zu achten ist.
- Für Praxisprojekte zu Technik & Alter(n) zeigt die STS auf, dass ältere Menschen nicht passive Nutzer*innen, sondern aktive Gestalter*innen von Technologien sind.

## Footnotes

1 Mit dem Begriff der „digitalen Technologien“ bezeichnen wir digitale Informations- und Kommunikationstechnologien, also einerseits Computer, Laptop, Smartphone und, quer dazu liegend, verschiedene Anwendungsbereiche des Internets, andererseits „smarte“ assistive Technologien aus dem AAL-Bereich.

## References

[1] Baars J (1991). The challenge of critical gerontology: the problem of social constitution. J Aging Stud.

[2] Bergschöld JM, Neven L, Peine A (2019). DIY gerontechnology: circumventing mismatched technologies and bureaucratic procedure by creating care technologies of one’s own. Sociol Health Illn.

[3] Bischof A, Hergesell J, Maibaum A, Meister M (2020). „Wir wollten halt etwas mit Robotern in Care machen“. Epistemische Bedingungen der Entwicklung von Robotern für die Pflege. Genese und Folgen der „Pflegerobotik“. Die Konstitution eines interdisziplinären Forschungsfeldes.

[4] Calasanti T, Biggs S, Lowenstein A, Hendricks J (2003). Theorising age relations. The need for theory: critical approaches to social gerontology.

[5] D21 Digital Index (2020) Jährliches Lagebild zur Digitalen Gesellschaft. https://initiatived21.de/app/uploads/2019/01/d21_index2018_2019.pdf. Zugegriffen: 16. Dez. 2020

[6] Ehlers A, Bauknecht J, Naegele G (2020). Abschlussbericht zur Vorstudie „Weiterbildung zur Stärkung digitaler Kompetenz älterer Menschen“.

[7] Endter C, Domínguez-Rué E, Nierling L (2016). Skripting age—the negotiation of age and aging in ambient assisted living. Ageing and technology.

[8] Felt U, Fouché R, Miller CA, Smith-Doerr L (2017). The handbook of science and technology studies.

[9] Fernández-Ardèvol M, Rosales A, Loos E, Peine A, Beneito-Montagut R, Blanche D, Östlund B (2019). Methodological strategies to understand smartphone practices for social connectedness in later life.

[10] Fischer B, Peine A, Östlund B (2019). The importance of user involvement: a systematic review of involving older users in technology design. Gerontologist.

[11] Greenhalgh T, Shaw S, Wherton J, Hughes G, Lynch J, A’Court C, Hinder S, Fahy N, Byrne E, Finlayson A, Sorell T, Procter R, Stones R (2016). SCALS: a fourth-generation study of assisted living technologies in their organisational, social, political and policy context. BMJ Open.

[12] Gómez DL (2015). Little arrangements that matter. Rethinking autonomy-enabling innovations for later life. Technol Forecast Soc Change.

[13] Haraway D (1985). Manifesto for cyborgs: science, technology, and socialist feminism in the 1980’s. Social Rev.

[14] Höppner G, Urban M (2018). Where and how do aging processes take place in everyday life? Answers from a new materialist perspective. Front Sociol.

[15] Jasanoff S (2004). States of knowledge: the co-production of science and social order.

[16] Joyce K, Loe M (2010). A sociological approach to ageing, technology and health. Sociol Health Illn.

[17] Klebbe R, Eicher C, Hergesell J, Maibaum A, Meister M (2020). Wer sind eigentlich diese Nutzer? Zur Rolle älterer und pflegebedürftiger Erwachsener in der Entwicklung robotischer Assistenzsysteme. Genese und Folgen der „Pflegerobotik“. Die Konstitution eines interdisziplinären Forschungsfeldes.

[18] Kolland F, Wanka A, Gallistl V, Künemund H, Vogel C, Schroeter K (2019). Technik und Alter – Digitalisierung und die Ko-Konstitution von Alter(n) und Technologien. Handbuch Soziologie des Alter(n)s.

[19] Lassen AJ, Moreia T (2020). New bikes for the old. Sci Technol Stud.

[20] Marshall B, Katz S (2018). How old am I? Digital culture and quantified ageing. Digit Cult Soc.

[21] Mitzner TL, Savla J, Boot WR, Sharit J, Charness N, Czaja S, Rogers WA (2019). Technology adoption by older adults: findings from the PRISM trial. Gerontologist.

[22] Moreia T (2016). Science, technology and the ageing society.

[23] Neven L (2010). ‘But obviously not for me’: robots, laboratories and the defiant identity of elder test users. Sociol Health Illn.

[24] Neven L, Peine A (2017). From triple win to triple sin: how a problematic future discourse is shaping the way people age with technology. Societies.

[25] Peine A, Neves B (2019). Technology and ageing—theoretical propositions from science and technology studies (STS). Ageing and digital technology.

[26] Peine A (2019). The co-constitution of health systems and innovation: comment on “What health system challenges should responsible innovation in health address? Insights from an international scoping review”. Int J Health Policy Manag.

[27] Peine A, Neven L (2019). From intervention to co-constitution: new directions in theorizing about aging and technology. Gerontologist.

[28] Peine A, Faulkner A, Jæger B, Moors E (2015). Science, technology and the ‘grand challenge’ of ageing—understanding the socio-material constitution of later life. Technol Forecast Soc Change.

[29] Pelizäus-Hoffmeister H, Birken T, Schweiger P, Sontheimer R (2018). Technik für ein selbstbestimmtes Leben im Alter. Eine Forschungsstrategie zur kontextintegrierenden und praxiszentrierten Bedarfsanalyse. Forum Qual Soc Res.

[30] Rammert W (2008). Technik und Innovationen. Kerninstitutionen der modernen Wirtschaft.

[31] Urban M (2017). “This really takes it out of you!” Senses and emotions in digital health practices of elderly. Digit Health.

[32] Wanka A, Gallistl V (2018). Doing age in a digitized world—a material praxeology of aging with technology. Front Sociol.

